# Comprehensive framework for thyroid disorder diagnosis: Integrating advanced feature selection, genetic algorithms, and machine learning for enhanced accuracy and other performance matrices

**DOI:** 10.1371/journal.pone.0325900

**Published:** 2025-06-18

**Authors:** Ankur Kumar, Sanjay Dhanka, Abhinav Sharma, Anchal Sharma, Surita Maini, Mochammad Fahlevi, Fazla Rabby, Mohammed Aljuaid, Rohit Bansal

**Affiliations:** 1 SCEE, IIT Mandi, Mandi, Himachal Pradesh, India; 2 Department of Electrical and Instrumentation Engineering, Sant Longowal Institute of Engineering and Technology, Longowal, Punjab, India; 3 Management Department, BINUS Online, Bina Nusantara University, Jakarta, Indonesia; 4 Operation Research & Management Sciences, Faculty of Business and Management, Universiti Sultan Zainal Abidin (UniSZA), Kuala Terengganu , Malaysia; 5 Department of Management, Stanford Institute of Management and Technology, Sydney, Australia; 6 Department of Health Administration, College of Business Administration, King Saud University, Riyadh, Saudi Arabia; 7 Department of Management Studies, Vaish College of Engineering, Rohtak, India; National University of Sciences and Technology, PAKISTAN

## Abstract

Thyroid hormones control crucial physiological activities, such as metabolism, oxidative stress, erythropoiesis, thermoregulation, and organ development. Hormonal imbalances may cause serious conditions like cognitive impairment, depression, and nervous system damage. Traditional diagnostic techniques, based on hormone level measurements (TSH, T3, FT4, T4, and FTI), are usually lengthy and laborious. This study uses machine learning (ML) algorithms and feature selection based on GA to improve the accuracy and efficiency of diagnosing thyroid disorders using the UCI thyroid dataset. Five ML algorithms-LR, RF, SVM, AB, and DT- were tested using two paradigms: (1) default classifiers and (2) hybrid GA-ML models- GA-RF, GA-LR, GA-SVM, GA-DT, and GA-AB. The data pre-processed included handling missing values, feature scaling, and correlation analysis. In this case, the performance metrics used for model evaluation are accuracy, F1 Score, sensitivity, specificity, precision, and Cohen’s Kappa with 80% of the dataset to train the model and the rest 20% used to test it. Among the non-hybrid models, RF achieved the highest accuracy, which was 93.93%. The hybrid GA-RF model outperformed all others, achieving a remarkable accuracy of 97.21%, along with superior metrics across all the evaluated parameters. These findings highlight the diagnostic potential of the GA-RF model in providing faster, more accurate, and reliable thyroid disorder detection. The research illustrated the potential of the hybrid GA-ML approaches to improving the clinical diagnostic process while proposing a strong and scalable approach towards thyroid disorder identification.

## Introduction

Thyroid disease is widespread among humans as one of the many conditions characterized by either a low or high activity of the thyroid, thereby leading to an imbalance in thyroid hormone production [1]. The thyroid hormone is produced by the thyroid gland, which among all the endocrine glands is the one to perform. The primary function of hormones is to fasten metabolism of the human body, direct calorie burning, and keep other hormonal glands in check when there’s excess secretion [2]. One in every ten Indians battle with the problem of thyroid disease, mainly attacking women aged from 17 to 54 years [3]. Disorders of the thyroid can hence lead to hypertension, hypercholesterolemia, cardiovascular disorders, infertility, and depression. In general, there are two hormones: triiodothyronine (T3) and thyroxine (T4), responsible for basal metabolism and maintaining the energy level in the body [4]. T3 and T4 secretion is controlled by Thyroid-stimulating hormone (TSH) released by the pituitary gland. The two most common thyroid diseases are hypothyroidism and hyperthyroidism. Hypothyroidism is the disorder in which the secretion from the thyroid gland is reduced and results in low T3 and T4 and therefore an increase in TSH levels. Symptoms of hypothyroidism include weight gain, fatigue, and cognitive impairment [5,6]. By contrast, hyperthyroidism is the disease whereby excessive hormones are secreted from the thyroid gland and result in increased levels of T3 and T4 and low levels of TSH. Symptoms of hyperthyroidism include hair loss, anxiety, and excessive sweating. Both conditions necessitate medical attention; treatment may involve hormone replacement therapy or medications intended to regulate thyroid hormone levels.

Regular check-ups and proper management can assist individuals afflicted with thyroid diseases in leading healthier lives [6,7]. Detecting thyroid diseases at an early stage is crucial to avoid severe complications, because of this, implementing preventive measures is essential. Various methods are employed to diagnose thyroid disorders, including clinical assessments, imaging examinations, blood tests and tissue biopsies. However, these approaches are not foolproof; the diagnosis of thyroid disorders through laboratory analysis is complex, requiring extensive expertise and experience from medical professionals. Although recent years have witnessed a surge in research studies that focus on the utilization of Machine Learning (ML) techniques to specifically detect various thyroid diseases, ML has demonstrated significant promise in the healthcare sector, showing better performance in addressing these challenges [7]. The advancement in computational power has further facilitated the implementation of complex and time-consuming ML algorithms in this domain. Consequently, ML is increasingly emerging as a significant tool for diagnosing and managing thyroid disorders effectively. ML algorithms (which are fundamentally computer-based statistical methodologies) can be trained to analyze extensive quantities of data. This enables the identification of recurring patterns. However, the intricacy of these algorithms poses challenges; they require large datasets to operate optimally. Although the potential is vast, it is imperative to approach their implementation with caution (because of the fact that) the outcomes can fluctuate considerably based on the quality of the data utilized.

Recent advancements continue to highlight the growing role of ML and AI in clinical diagnostics and healthcare. Studies have shown that ML algorithms can achieve high diagnostic accuracy in identifying complex medical conditions, including hematologic disorders [8]. Additionally, AI techniques are proving instrumental in areas such as drug discovery and the fight against antimicrobial resistance, where they support tasks like high-throughput screening, compound optimization, and drug repurposing [9,10]. These developments underscore the transformative potential of AI in improving diagnostic efficiency, accelerating therapeutic development, and advancing personalized medicine. This empowers doctors to simultaneously consider multiple patient characteristics using ML algorithms for improved diagnosis and patient identification [11,12]. This research study explored the use of ML classifiers to detect and classify hypothyroidism using logistic regression (LR), random forest (RF), support vector machine (SVM), AdaBoost (AB), and decision tree (DT) algorithms. The models are used to predict and classify thyroid conditions into two classes, i.e., normal and hypothyroidism. The performance of the models is evaluated in terms of accuracy, sensitivity, specificity, F1 score, and Kappa statistic. The main contributions of this research study are as follows:

- A proper investigation of data of the patients, even including levels of thyroid hormones, demographics, and symptoms, is carried out to know which feature have to be included for a proper classification. “Mean Imputation” method was meant to fill the missing values, Pearson’s correlation coefficient was used to see the level of correlation of features and target, and “Standard Scalar” method was meant to have all the features on similar scales, that may better the performance of models in some cases.
- A thorough investigation of five multi-objective ML models, including LR, RF, SVM, AB, and DT along with default as well as hyperparameter tuning is conducted for the selection of the best model for the classification of thyroid disease.
- An integration of the meta-heuristic technique “Genetic Algorithm (GA)” with all the five multi-objective models mentioned here is utilized for the purpose of optimizing the inner hyperparameters of the models along with being a feature selector to determine the most potent features of the dataset along with hyperparameters.
- All the proposed models were validated by advanced technique such as “Stratified K-Fold Cross-Validation (CV)”. This ensures the model’s generalizability and avoids overfitting to the training data. Finally, the performance of the proposed model framework should be compared with existing state-of-the-art models to assess its relative merits and identify potential areas for future research.

### State-of-the-art

In this section, the authors provide a concise overview of the existing research on the use of ML techniques for identifying thyroid dysfunction. It is important to note that there is a limited number of systematic reviews or surveys on this specific topic in the current literature such as Alyas et al. [13], employed several ML algorithms, including RF, Artificial Neural Networks (ANN), DT, and K-Nearest Neighbors (KNN), to identify hyperthyroidism. Among these models, the RF algorithm emerged as the top performer by achieving the highest prediction accuracy of 94.8%. Guleria et al. [1] utilized ML and Deep Learning (DL) techniques for the early detection of thyroid disorders, using WEKA in Java. They employed ML algorithms like DT, SVM, and ANN, along with DL methods such as Convolutional Neural Networks (CNN) and Recurrent Neural Networks (RNN). Their deep learning-based ANN achieved an impressive accuracy rate of 93.82%, outperforming the RNN. Zhang et al. Mir et al. [14], distinguished between pathological and serological parameters of thyroid disease, consulting an endocrine specialist for guidance. They created predictive models for thyroid disease diagnosis based on these parameters separately and in combination. In the first experiment, they found that the bagging classification algorithm performed best when considering all dataset features, achieving an impressive accuracy of 98.56%. In the second experiment, focusing on serological features, the J48 classification algorithm proved most effective, with an accuracy of 92.07%. Keles et al. [4], emphasized the significant progress in AI-driven expert systems within the medical domain and underscored their readiness for targeted real-world applications. A particular note is that the “Expert System for Thyroid Disease Diagnosis (ESTDD),” showcases an impressive diagnostic accuracy of 95.33% for thyroid diseases.

Yeh et al. [15], presented work puts forth a novel rule-based classifier design methodology specifically, an enhanced version of Simplified Swarm Optimization (SSO) aimed at mining a dataset pertaining to the thyroid gland, which has been extracted from the UCI databases. This proposed methodology incorporates an elite concept intended to augment solution quality; for instance, it employs Close Interval Encoding (CIE) to effectively encapsulate the rule structure. Additionally, it utilizes the Orthogonal Array Test (OAT) to mitigate the risk of overfitting the training dataset. The computational outcomes yielded by this classifier surpass those of traditional classifiers documented in the literature, including but not limited to the Bayes classifier, K-Nearest Neighbors (KNN), k-Means clustering and 2D Self-Organizing Maps (2D-SOM). Furthermore, the classifier in question demonstrates superior performance relative to soft computing-based classifiers, such as the basic SSO, Genetic Algorithms (GA) and Immune Estimation of Distribution Algorithms (IEDA). However, it is crucial to recognize that while these advancements are significant, the field continues to evolve and further research is warranted to explore additional enhancements.

Zhang et al. [16] proposed a novel DL based methodology for the automation of thyroid disease detection presents a significant advancement, particularly in terms of diagnostic accuracy and efficiency when juxtaposed with traditional, subjective clinical assessments. The articulated multi-channel convolutional neural network (CNN) architecture has yielded a remarkable accuracy of 0.909 ± 0.048, alongside a precision of 0.944 ± 0.062, a recall of 0.896 ± 0.047, a specificity of 0.994 ± 0.001 and an F1 Score of 0.917 ± 0.057. Butchi Raju et al. [17], applied ML models on the hypothyroid dataset taken from the UCI data repository to partially fulfil thyroid disorder detection. The detection methods are based on pattern recognition, ML, and data mining techniques. The work presented in this article has shown that the F1 score of the AdaBoost model is a reliable one for predicting the classification of thyroid patients. Gupta et al. [18] presented a differential evolution (DE) for ML model optimization and leverages conditional generative adversarial networks for data augmentation. This study investigated the effectiveness of this approach through a series of experiments with as well as without model optimization and achieved a superior accuracy with AdaBoost and DE optimization method.

Recent advancements in medical imaging and ML have significantly improved diagnostic accuracy across various conditions. Khan et al. [19] demonstrated the effectiveness of the XG-Ada-RF ensemble model for brain tumour classification in MRI images, achieving high accuracy rates of 94.9% for healthy cases and 95.9% for tumour cases. Anand et al. [20] explored various architectures, including VGG19 and CNNs, achieving accuracy rates up to 98.16% and underscoring the potential of ensemble methods and hybrid models in enhancing diagnostic performance. Khan et al. [21] introduced a novel approach combining Least Squares Support Vector Machines (LS-SVM) with Multi-Scale Morphological Texture Features (MMTF) from T1-weighted MRI images, achieving an impressive 98.97% accuracy with no False Positives. Singh et al. [22] achieved a 95.99% accuracy in COVID-19 detection from chest CT images using a feature selection technique that reduced the initial 213 features, with XGBoost emerging as the top-performing classifier. Khanna et al. [23] emphasized the importance of feature selection (FS) in computer-aided diagnosis (CAD) systems, achieving a 97.96% accuracy in classifying benign and malignant tumours using Teaching Learning-Based Optimization (TLBO), Elephant Herding Optimization (EHO), and their hybrid. Singh et al. [24] proposed a Gravitational Search Optimization Algorithm (GSOA) for glaucoma detection, achieving a 95.36% accuracy with selected features from retinal fundus images. Singh et al. [25] explored three metaheuristic feature selection strategies—Bacterial Foraging Optimization Algorithm (BFOA), Emperor Penguin Optimization (EPO), and a hybrid of both (hBFEPO)—achieving outstanding results with 100% precision and specificity, 98.49% accuracy, and 99.60% AUC in breast cancer classification. Singh et al. [26] proposed two novel two-layered approaches—BA-BCS and BCS-PSO—using Particle Swarm Optimization (PSO), Binary Cuckoo Search (BCS), and Bat Algorithm (BA) for glaucoma detection, achieving up to 98.95% accuracy, thereby providing efficient solutions for enhancing diagnostic performance and aiding medical professionals. The literature survey conducted involved the application of various ML algorithms and diverse data preprocessing techniques on the thyroid dataset, resulting in promising outcomes in terms of accuracy, precision, F1 score, sensitivity, and other performance metrics. While the current results are promising, there is room for improvement. Specifically, refining data preprocessing techniques like scaling and handling missing values, along with exploring advanced methods like ensemble techniques and addressing class imbalance, could lead to even better outcomes in future research. The literature survey of several additional studies is listed in Table 1.

**Table 1:** Additional literature survey of the thyroid disease.

References	Year	Dataset	Preprocessing/Feature Selection	Classifiers	Accuracy (%)	Limitations of the study
[14]	2020	UCI	Normalization	SVM	92.07	Limited dataset size; lacks testing with other classifiers for comparison.
[16]	2022	Tertiary Hospital	Data Scaling	CNN	90.90	Dataset is domain-specific; results may not generalize to other populations.
[17]	2021	UCI	---	LR	88.80	No feature selection or preprocessing; lower accuracy compared to others.
[27]	2018	UCI	Missing value handled	K-NN	96.90	Relies on imputed data, which may introduce bias or inaccuracies.
[28]	2020	UCI	Correlation	SVM	92.92	Limited focus on feature selection techniques beyond correlation analysis.
[29]	2022	UCI	Normalization	ANN CatBoost XGBoost	95.87 95.38 95.33	Lacks exploration of hybrid models; high computational cost for ensemble models.
[30]	2022	UCI	---	LR	81.25	No preprocessing or feature selection; significantly lower accuracy observed.

As per mentioned literature and Table 1 success of data mining techniques in predicting thyroid disorders greatly depends on choosing the right features and using suitable ML algorithms. Finding the best combination of important features and the most effective algorithm with proper inner parameter tuning is essential. This research seeks to identify the most efficient data mining techniques and feature sets for predicting thyroid disorders. However, existing methods are often inadequate, highlighting the need for extensive evaluation and experimentation to determine the most accurate techniques and features.

## Material and methodology

**Fig 1:**
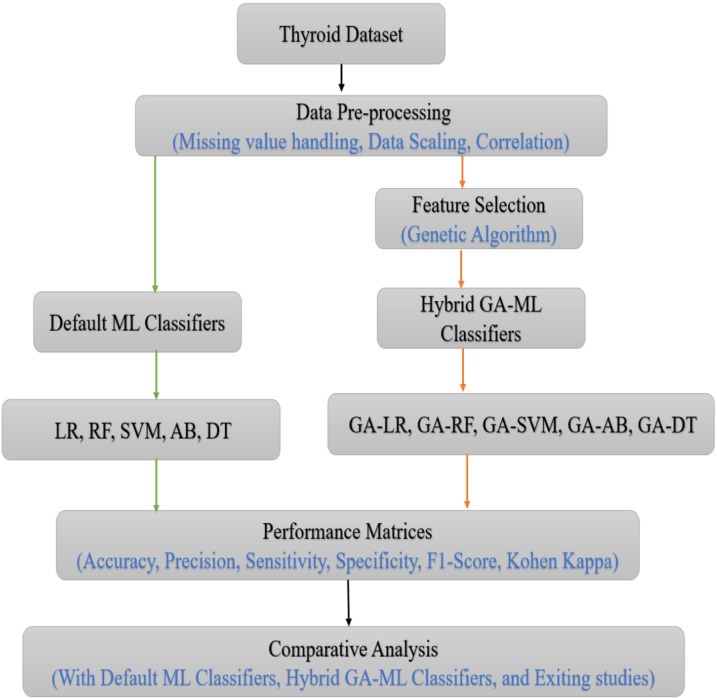
Workflow chart of the proposed study.

In this study, the authors performed several preprocessing steps, such as addressing missing data, scaling the data, and assessing variable correlations. Following this preprocessing, they applied an advanced meta-heuristic GA technique for feature selection in conjunction with five standard ML algorithms such as LR, RF, SVM, AdaBoost, and DT on the UCI thyroid dataset. The study comprised two experiments, first with default ML classifiers and second with hybrid GA-ML, and evaluated model performance using various metrics including Accuracy, F1 Score, Specificity, Sensitivity, Cohen’s Kappa, and Precision. The proposed methodology flowchart is illustrated in Fig 1.

### Data description

In this research work, the authors selected the UCI Sick-euthyroid dataset [31] over the other ten UCI thyroid datasets primarily because of its larger size and unique class distribution. The availability of a large dataset allows us to develop more advanced and insightful applications. The Sick-euthyroid dataset contains 3,772 rows and 31 data columns, making it a valuable resource for our research. The other datasets each contain 2,800 instances without any missing values. Additionally, both datasets share the same number of instances, with 972 cases included in their respective test sets. Despite their relatively smaller size and complete data, authors still opted for the Sick-euthyroid dataset due to its potential for discovering new insights and the fact that it has not been extensively studied by many researchers before. The author’s focus on the Sick-euthyroid dataset allowed us to delve deeper into its characteristics and analyze the results comprehensively. By studying this dataset thoroughly, the authors aim to assist future researchers in predicting similar types of multiclass thyroid datasets, enabling further advancements in the field. Table 2 outlines the data set’s general structure.

**Table 2:** Description of the dataset with attributes.

S. No.	Attributes	Categorical Value		Missing Values
1	Age	Continuous		Yes
2	Sex	M	F	Yes
3	On_thyroxine	f	t	No
4	Query_on_thyroxine	f	t	No
5	On_antithyroid_medication	f	t	No
6	Thyroid_surgery	f	t	No
7	Query_hypothyroid	f	t	No
8	Query_hyperthyroid	f	t	No
9	Pregnant	f	t	No
10	Hypopituitary	f	t	No
11	Psych	f	t	No
12	Thyroid surgery	f	t	No
13	I13_Treatment	f	t	No
14	Sick	f	t	No
15	tumor	f	t	No
16	lithium	f	t	No
17	Goitre	f	t	No
18	TSH_measured	n	Y	No
19	TSH	Continuous		Yes
20	T3_measured	n	Y	No
21	T3	Continuous		Yes
22	TT4_measured	n	Y	No
23	TT4	Continuous		Yes
24	T4U_measured	n	Y	No
25	T4U	Continuous		Yes
26	FTI_measured	n	Y	No
27	FTI	Continuous		Yes
28	TBG_measured	n	Y	No
29	TBG	Continuous		Yes
30	Referral_source	Continuous		No
31	Thyroid	f	t	No

### Data refining

**Fig 2:**
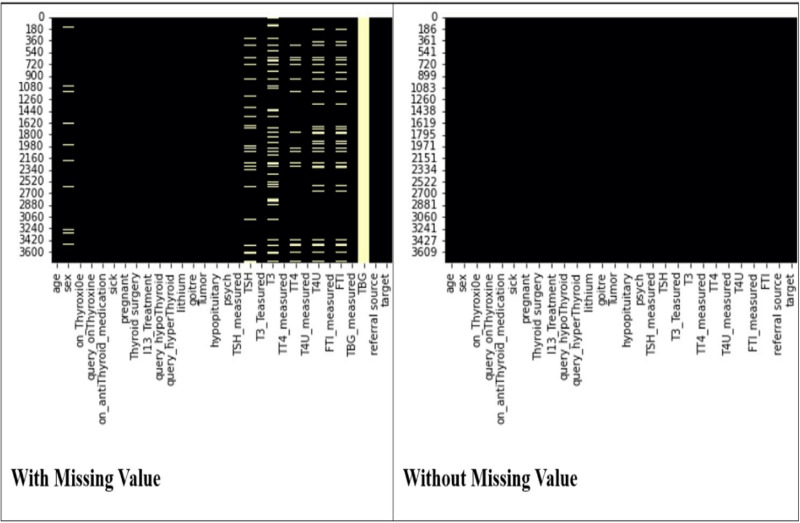
Missing value imputation.

At the beginning of data pre-processing, an analysis was conducted to examine the potential values associated with each characteristic. Upon noticing that the TBG column contains a substantial 91.78% missing value, the decision was made to eliminate this column from consideration. To address the remaining missing values in various columns or features, the authors employed “Mean Imputation” to replace them as shown in Fig 2. Subsequently, a transformation was applied to convert object data types into integers. This was achieved by assigning numerical equivalents to specific values: ‘t’ was replaced with 1, ‘f’ with 0, ‘Y’ with 1, ‘n’ with 0, ‘Thyroid’ with 1, ‘negative’ with 0, ‘F’ with 1, and ‘M’ with 0. In a dataset with substantial class imbalance, the distribution of data across each class varies significantly. Addressing this imbalance is crucial, this is particularly relevant in fields like medical analysis where incorrectly identifying a patient with Thyroid as healthy (a false negative) carries greater consequences than incorrectly diagnosing a healthy individual as having Thyroid (a false positive). A false negative could lead to a poor decision, making it far more detrimental than a false positive.

### Correlation and normalization

**Fig 3:**
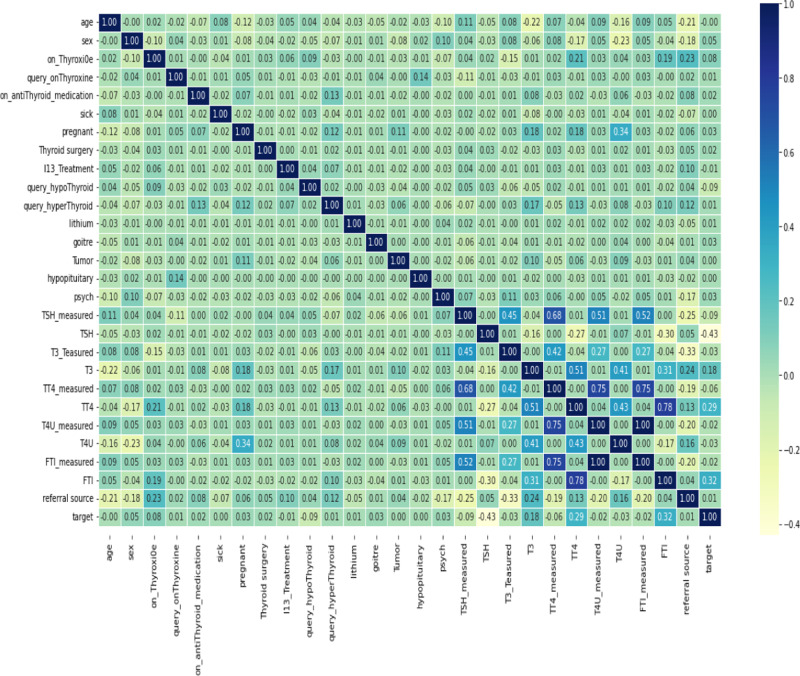
Correlation between features and target.

The most widely recognized correlation measure in statistics is the “Pearson Correlation Coefficient” [32]. The Pearson product-moment correlation coefficient, commonly referred to as Pearson’s correlation, is a metric that quantifies the strength and direction of the relationship between two variables measured on at least an interval scale. A stronger relationship between the variables results in a Pearson correlation coefficient, denoted as ‘r,’ that is closer to either +1 or −1, depending on whether the relationship is positive or negative, respectively as shown in Fig 3. When the correlation coefficient reaches +1 or −1, it signifies that all the data points precisely align with the best-fit line. In other words, achieving towards value of +1 implies a perfect positive linear relationship, as ‘FTI’ gone maximum toward positive, where an increase in one variable corresponds to a proportional increase in the other, while a value of −1 implies a perfect negative linear relationship, as ‘TSH’ gone maximum towards negative, where an increase in one variable corresponds to a proportional decrease in the other.

**Fig 4:**
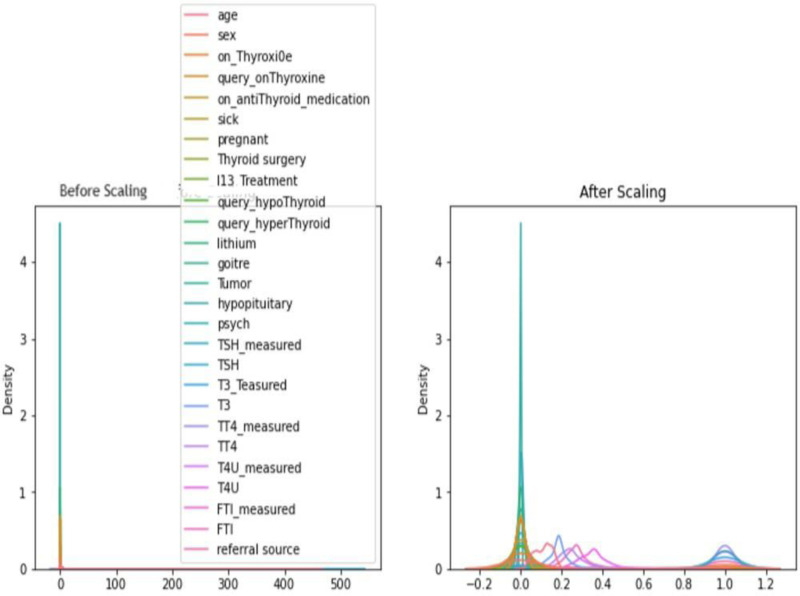
Data scaling visualization.

After calculating the Pearson correlation to assess the linear relationships between variables, the authors applied the “Standard scaler” to the data to ensure that the data was scaled or normalized for further analysis or modelling purposes. Scaling involves rescaling the data to a common range or distribution to prevent any variables from dominating the analysis due to differences in their units or magnitudes. Fig 4 provides a graphical representation of the process of data scaling using a kernel density estimate (KDE) plot [33].

### Algorithms overview

#### Decision tree (DT).

DT technique is supervised ML which involves division of continuous data into subsets by specified parameters. In this technique, every internal node in the decision tree is an attribute and every branch represents a decision rule [34]. The outcome at each leaf node is interpreted recursively. It applies Top-Down approach starting from root and sorts of data in a way that reaches the leaf node. The training data is divided into subsets using criteria such as Gini Index, Information Gain, Entropy, Gain Ratio, and Chi-Square. In most datasets, the Gini Index and Information Gain are used to measure the quality of the splits using equations 1 and 2. The decision tree algorithm continues to split each child tuple until all tuples belong to the same class and no more attributes are needed for further classification. The Gini Index is used to improve precision and diagnostic accuracy in the process. Overall, decision trees prove to be a powerful means of classification tasks in the field of ML because such models are interpretable, easy to understand, and can handle both categorical and continuous data. By recursively partitioning the data based on attribute values, decision trees can solve complex problems efficiently and make accurate predictions [35].


$$\begin{array}{c} Information\;Gain=Entropy(S)[(Weighted\;Avg)\times Entropy(each\;feature) \end{array}$$ (1)

$$\begin{array}{c} Gini\;Index=1-\sum_{j=1}^{c}P^{2}j\; \end{array}$$ (2)

Here in Eq. (2), Pj is proportion of the samples that belongs to class c for a particular node.

#### Random forest (RF).

RF is a ML based ensemble technique whereby numerous independent decision trees form ensembles that work collaboratively [29]. That means one tree is based upon one subset of data produced with some kind of randomized sampling. Then, to derive a final prediction score, these ensembles of random forest gather their predictions from various single decision trees through techniques that most commonly use voting and/or averaging. The important features within the dataset can be identified through random forests. Feature importance analysis provides a simple indicator of the significance of each feature in contributing to the predictive performance of the model. Feature selection is generally used in classification studies in order to improve accuracy and simplify the data representation. Different approaches are used in feature selection. These can be performed by filtering techniques, and others by wrapper techniques. Filtering methods perform feature selection without the knowledge of any classification algorithm; it just depends on the characteristics of the dataset. The utility of the feature is analyzed through an individual function measuring the relevance and contribution to the whole model accuracy [29]. RF generally produces more reliable ensemble predictions compared to a single DT. The basic difference between RF and DT is that RF uses a bagging of multiple decision trees, which helps to decrease overfitting and enhances generalization. For every feature, the ${fi}_{i}$ value of a test statistic is computed using equation 3 in the feature selection procedure. The ${fi}_{i}$ value quantifies the significance of a feature in the context of the model and helps in identifying which features are the most important to predict correctly.


$$\begin{array}{c} norm\;{fi}_{i}=\frac{{fi}_{i}}{\sum g_{ij\in \;all\;features}^{.}fi} \end{array}$$ (3)

Here in Eq. (3), norm ${fi}_{i}$ is the normalized importance of feature i, ${fi}_{i}$ represents the importance of feature i.

#### AdaBoost (AB).

AdaBoost, short for Adaptive Boosting, is a supervised ensemble ML technique that aims to improve the performance of weak learners, usually decision trees, by iteratively emphasizing the misclassified instances from previous iterations. In each iteration, AdaBoost assigns higher weights to misclassified samples, allowing subsequent weak learners to focus on these challenging cases. The final prediction is obtained by combining the weighted outputs of individual weak learners, thus creating a strong ensemble model that can generalize better and have a higher accuracy than a single weak learner as depicted in equation 4. AdaBoost’s ability to adapt to different learning algorithms and its effectiveness in dealing with complex datasets make it an advanced algorithm in the field of ML [36].


$$\begin{array}{c} minimize=\sum {\mathrm{\backslash nolimits}}_{i}w_{i}*I\;\left(y_{i}\neq \;h_{i}\left(x_{i}\right)\right) \end{array}$$ (4)

Where, $w_{i}$ is weight associated with the $i^{th}$ training example, $h_{i}\left(x_{i}\right)$ is prediction of the $i^{th}$ weak learner for the $i^{th}$ training example, and $I\;\left(y_{i}\neq \;h_{i}\left(x_{i}\right)\right)$ is an indicator function that equals one if the prediction is incorrect and zero otherwise.

#### Logistic regression (LR).

LR is a general form of model based on the generalized linear regression analysis and mostly used as a supervised learning algorithm, which can be used in regression, binary classification, and multi-class problems. There are three fundamental steps involved in the process of LR: define the prediction function, formulate a loss function, and come up with regression parameters such that they minimize this loss function. In the application of LR to either regression or classification tasks, a cost function is first defined. The objective is to find the optimal model parameters using an iterative optimization technique. This iterative process fine-tunes the parameters to reduce the cost function. Finally, the quality of the model is evaluated to assess its performance. In summary, LR is a strong approach used in different applications, and its application includes defining prediction functions, designing loss functions, optimizing parameters, and evaluating the effectiveness of the model [37]. The prediction function is associated with the Sigmoid function which is defined in equation 5.


$$\begin{array}{c} S(x)=\frac{1}{1-e^{-x}} \end{array}$$ (5)

#### Support vector machine (SVM).

SVM is one of the supervised learning models. It is used for both classification and regression tasks. The goal of SVM is to find the optimal hyperplane in the feature space that maximizes the distance between classes. Data points closest to this hyperplane are called support vectors that decide the decision boundary of a model. SVMs can handle the classification of linear and non-linear problems. It can leverage kernel functions to map feature space to higher dimensions where linear separation might be easily discovered. The regularization parameter (C) in SVM controls the trade-off between maximizing the margin and minimizing misclassifications as shown in equations 6 and 7. SVMs are widely applied across various domains due to their effectiveness in complex and high-dimensional datasets, such as text classification and image analysis, offering robust performance and good generalization capabilities [37].


$$\begin{array}{c} minimize=\;\frac{1}{2}{\left\|w\right\|}^{2} \end{array}$$ (6)

$$\begin{array}{c} subject\;to=\;y_{i}\left(w^{T}*x_{i}+b\right)\geq 1,\;for\;i=1,\ldots ,n \end{array}$$ (7)

Where, w is weight vector that defines the hyperplane, b is bias term that shifts the hyperplane, $x_{i}$ is feature vector of the $i^{th}$ data point, and $y_{i}$ is class label of the $i^{th}$ data point.

#### Genetic algorithm (GA).

**Fig 5:**
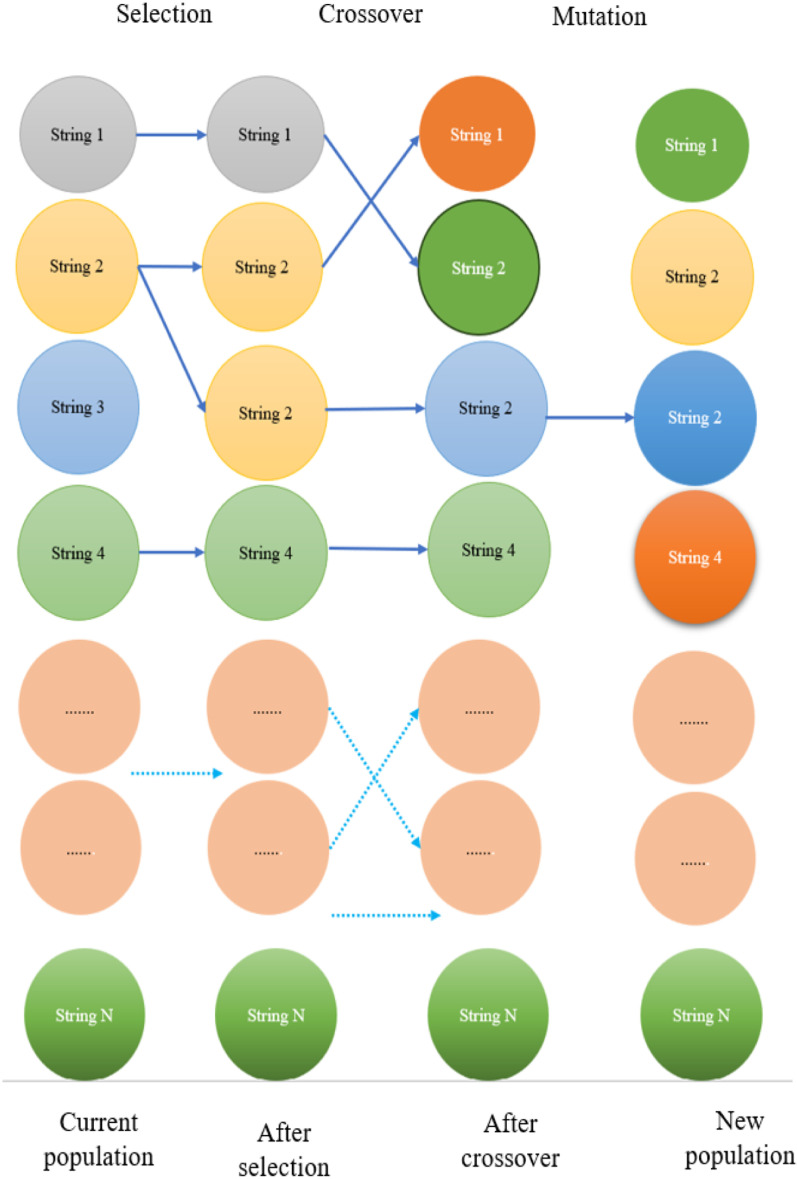
The basic operations of GA approach.

GA simulates the process of natural selection to solve optimization problems. In each generation, the selection phase identifies individuals (represented as strings of genetic information) based on their fitness, typically determined by a problem-specific objective function. Higher-fitness individuals are more likely to be chosen as parents for the next generation. The recombination phase involves applying crossover, where genetic material from selected parents is exchanged to create offspring [38]. Various crossover methods dictate how genetic information is combined. Mutation, which introduces random changes to individual chromosomes, can further diversify the population. This iterative process of selection, crossover, and mutation as shown in Fig 5 continues over multiple generations, aiming to evolve a population towards increasingly optimal solutions in complex search spaces where traditional methods may be inefficient [39,40].

### Proposed GA-ML approaches

In the current GA approach, most of the previous articles represent a combination of attributes from a set of 30 (corresponding to the 30 attributes in the dataset). This necessitates a large population size of 2^30^ individuals to cover all possible attribute combinations. To improve the GA’s performance, a strategy is implemented where attributes containing critical information (such as TSH, TT4, T4U, hypopituitary, thyroid_surgery, and FTI) are assigned a lower mutation probability (specifically, 10^−3^). Conversely, the remaining attributes have a higher mutation probability, promoting the exploration of diverse individuals with potentially higher fitness. In the simulation using the thyroid dataset, two sets of attributes (TSH, TT4, T4U, hypopituitary, thyroid_surgery, and FTI) and (TBG, Goitre, lithium, and tumour) are identified as most significant and hence are subjected to the lower mutation probability of 10^−3^.

**Fig 6:**
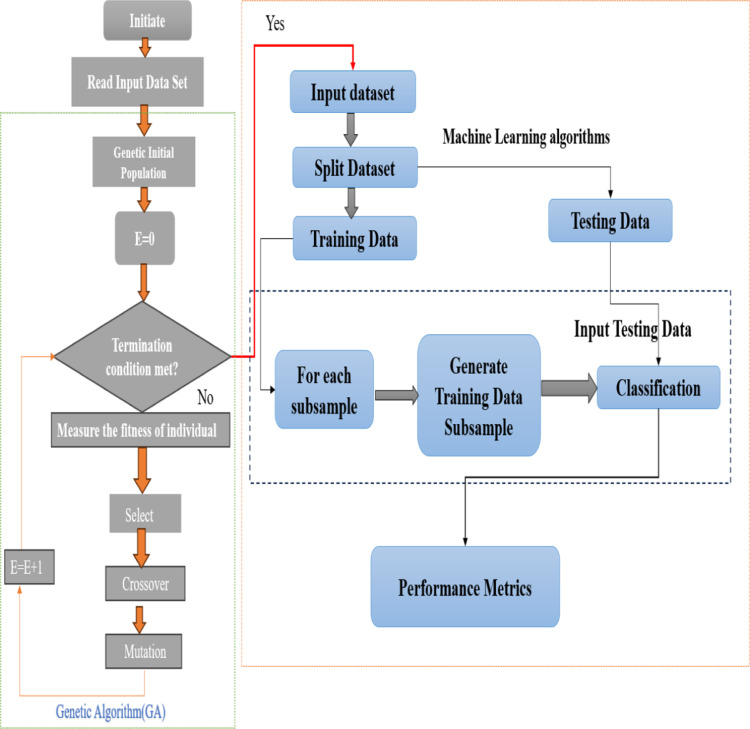
Proposed hybrid GA- ML algorithms.

Furthermore, to incorporate this mutation strategy into the GA, the initialization of the population is adapted. At the start of the GA, individuals in the initial population are deliberately configured to include the most significant attributes identified, ensuring that everyone begins with this critical information. GA operates through four core steps: modified selection, crossover, modified mutation, and fitness calculation. This approach acknowledges that even non-fit individuals (rejected chromosomes) may harbour valuable genetic material that can guide the search process toward promising areas of the solution space. During selection, the primary objective is to identify the fittest individuals based on their fitness values, which are determined using a ML algorithm. ML plays a crucial role by efficiently evaluating the model’s fitness, thereby influencing its survival into the next generation within the GA framework. ML algorithms are particularly effective in mitigating overfitting, a common challenge in thyroid disease prediction tasks. In the proposed hybrid GA-ML approach, ML classifiers are integrated with GA to conduct feature selection. This integration capitalizes on the ensemble-based nature of ML algorithms. Fig 6 presents a detailed overview of the hybrid GA-ML approach, illustrating the synergy between GA and ML for feature selection and model optimization. ML algorithms, renowned for their ability to create ensembles of individual learners like decision trees, prove highly effective in real-world applications. Table 3 pseudocode further insight into the specific ML algorithm employed within this hybrid framework. Out of the 30 features, 23 were selected by the GA approach based on their selection criteria, which include mutation and other genetic operations, as listed in Table 4.

**Table 3:** Proposed GA-ML algorithms pseudocode.

1. Initialize Population: - Randomly generate initial populations of binary-encoded chromosomes representing feature selection (e.g., [1, 0, 1, 0, 1] where 1 indicates inclusion of a feature and 0 indicates exclusion). 2. Define Fitness Function: - Define a fitness function that evaluates the performance of an ML model using the selected features. This could be based on metrics like accuracy, F1-score, or other relevant measures. 3. Genetic Algorithm Loop: 4. repeat { 5. Selection: - Evaluate fitness of each chromosome in the population using the defined fitness function. - Select chromosomes from the population as parents for crossover based on their fitness (e.g., using roulette wheel selection). 6. Crossover: - Apply crossover to pairs of selected parents to generate offspring (new chromosomes). - Use a crossover method (e.g., single-point crossover, two-point crossover) to exchange genetic material between parents and create new feature combinations. 7. Mutation: - Apply mutation to introduce variability into the population. - Randomly flip bits (features) in chromosomes to explore new feature combinations. 8. Evaluate Offspring: - Evaluate the fitness of newly generated offspring using the fitness function. 9. Survivor Selection: - Combine the parent and offspring populations. - Select individuals to form the next generation (e.g., using elitism or tournament selection). 10. } until termination criteria met (e.g., maximum number of generations, convergence of fitness). 11. Select Best Features: - After the GA loop completes, select the best-performing chromosome (feature combination) from the final population based on the fitness function. 12. Train ML Model with Selected Features: - Use the selected features to train an ML model (e.g., SVM, LR) on a training dataset. 13. Evaluate Model Performance: - Evaluate the performance of the trained model using the selected features on a separate validation or test dataset. 14. Output: - Return the selected features and the performance metrics of the ML model.

**Table 4:** Selected features by GA approach.

S.No.	Attributes	S.No.	Attributes
1.	Age	13.	Goitre
2.	Sex	14.	TSH_measured
3.	On_thyroxine	15.	TSH
4.	on_antithyroid_medication	16.	T3_measured
5.	thyroid_surgery	17.	T3
6.	query_hypothyroid	18.	TT4_measured
7.	query_hyperthyroid	19.	TT4
8.	lithium	20.	T4U_measured
9.	hypopituitary	21.	T4U
10.	Thyroid surgery	22.	FTI_measured
11.	I13_Treatment	23.	FTI
12.	tumor		

### Performance metrics

Performance metrics are quantitative measures that objectively assess and monitor the effectiveness of ML models. They offer a range of performance indicators that aid in comparing different ML algorithms. In this research work, the focus will be directed toward performance metrics commonly employed to address classification challenges. The authors can leverage performance metrics for categorization tasks, including Accuracy, Precision, Recall, F1-score, specificity, and Cohen’s kappa (K). Cohen’s kappa coefficient is a valuable metric that quantify the level of agreement between the model’s predictions and the ground truth labels, taking into account the possibility of random agreement. Values less than or equal to zero indicate no agreement between models. Values between 0.01 and 0.20 suggest slight or no agreement. Fair agreement is indicated by values between 0.21 and 0.40, while moderate agreement ranges from 0.41 to 0.60. Kappa values between 0.61 and 0.80 reflect substantial agreement, and values between 0.81 and 1.00 indicate almost perfect agreement. Table 5 presents the evaluation of various performance metrics using the Confusion matrix, which includes True Positive (TP), False Positive (FP), True Negative (TN), and False Negative (FN) [41–43].

**Table 5:** Performance metrics evaluation criteria.

S. No	Performance Metrics	Formula
1	Accuracy	$\frac{\left(TN+TP\right)}{(FP+TP+FN\;+TN\;}$
2	F1 Score	$\frac{2TP}{\left(2TP\;+\;FP\;+\;FN\right)}$
3	Precision	$\frac{TP}{(FP+TP}$
4	Sensitivity (Recall/TPR)	$\frac{TP}{\left(TP+FN\;\right)}$
5	Specificity	$\frac{TN}{TN+FP}$
6	Cohens kappa (K)	$\frac{2(TP\times TN-FN\times FP)}{\left(TP+FP)(FP+TN)+(TP+FN)(FN+TN\right)}$

### Experimental results with discussion

The experiment was conducted on a computer running the Windows 11 operating system equipped with a 1.8 GHz Intel Core i5 processor, 8 GB of RAM, Python, Jupyter notebook, pandas, NumPy, Matplotlib, and Seaborn. GA was employed for feature selection in experiment 2, narrowing down the total number of features from 30 to 23 based on GA’s selection criteria. Subsequently, the performance of default ML models was compared with those utilizing GA-selected features (GA-ML). To ensure robust evaluation, all ML models underwent validation using Stratified K-Fold cross-validation.

#### Experiments 1: default ML models.

**Fig 7:**
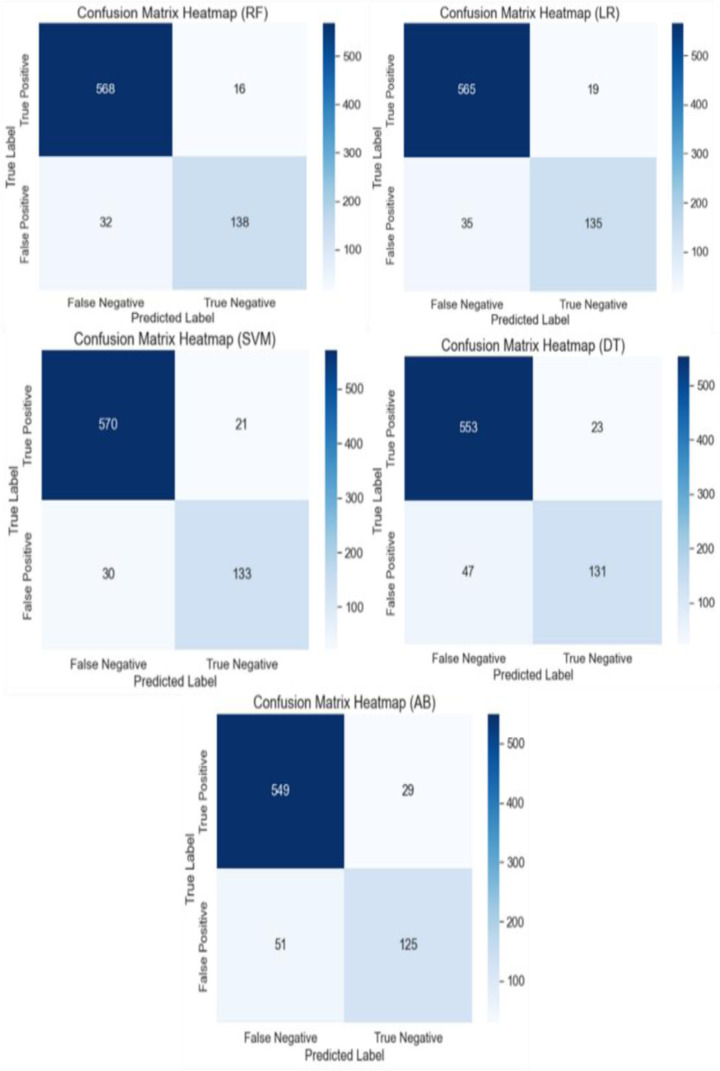
Confusion matrix of default ML classifiers.

**Fig 8:**
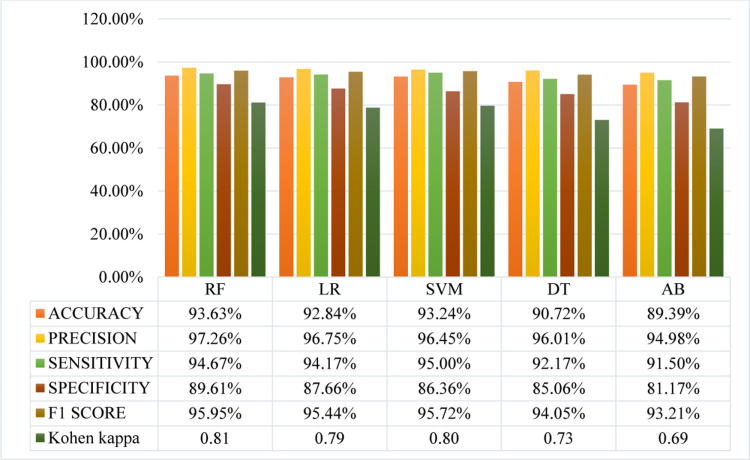
Performance metrics of default ML classifiers.

In the first experiments all the ML algorithms with default parameters, i.e., RF, LR, SVM, DT, and AB have trained on the 80% (3017 number of patients) data and subsequently tested on the remaining 20% (754 number of patients) data. The confusion matrix as well as performance matrices have been analyzed at this training-testing ratio, shown in Figs 7 and 8 respectively. From Fig 7, it was observed that the RF algorithm classified 568 patients’ data as true positive (TP), 138 patients’ data as true negative (TN), 32 patients’ data as false negative (FN), and 16 patients’ data as false positive (FP). Similarly, the confusion matrix results for all the algorithms have been obtained in the same manner. Afterward, the performance metrics of all the default models were analyzed at this training set in terms of accuracy, precision, sensitivity, specificity, f1 score, and K (Kappa values).

Fig 8 presents performance metrics for default ML models RF, LR, SVM, DT, and AB evaluated on the thyroid dataset. Among the models, RF demonstrates the highest overall performance with an accuracy of 93.63%, precision of 97.26%, sensitivity of 94.67%, specificity of 89.61%, an F1 score of 95.95%, and a Cohen’s Kappa of 0.81. This indicates RF’s strong ability to correctly identify positive cases while maintaining a balance between precision and recall. SVM follows closely with an accuracy of 93.24%, a precision of 96.45%, and the highest sensitivity of 95.00%, showing it effectively identifies true positives. However, its specificity is slightly lower at 86.36%, which means it has a slightly higher rate of false positives compared to RF. The F1 score of 95.72% and a Cohen’s Kappa of 0.80 suggest it is a strong performer overall. LR also performs well, with an accuracy of 92.84%, precision of 96.75%, and sensitivity of 94.17%. Its specificity is higher than SVM at 87.66%, resulting in an F1 score of 95.44% and a Cohen’s Kappa of 0.79, indicating good overall performance with a slight edge in precision. DT shows reasonable performance with an accuracy of 90.72%, precision of 96.01%, and sensitivity of 92.17%. Its specificity is 85.06%, F1 score is 94.05% and Cohen’s Kappa is 0.73, suggesting it is effective but less balanced compared to RF, SVM, and LR. AB has the lowest performance metrics among the compared models, with an accuracy of 89.39%, precision of 94.98%, and sensitivity of 91.50%. Its specificity is the lowest at 81.17%, resulting in an F1 score of 93.21% and a Cohen’s Kappa of 0.69. This indicates that AB has a higher rate of false positives and is less reliable compared to the other models. In summary, RF stands out as the top-performing model, followed closely by SVM and LR, while DT and AB show relatively lower performance in comparison.

**Fig 9:**
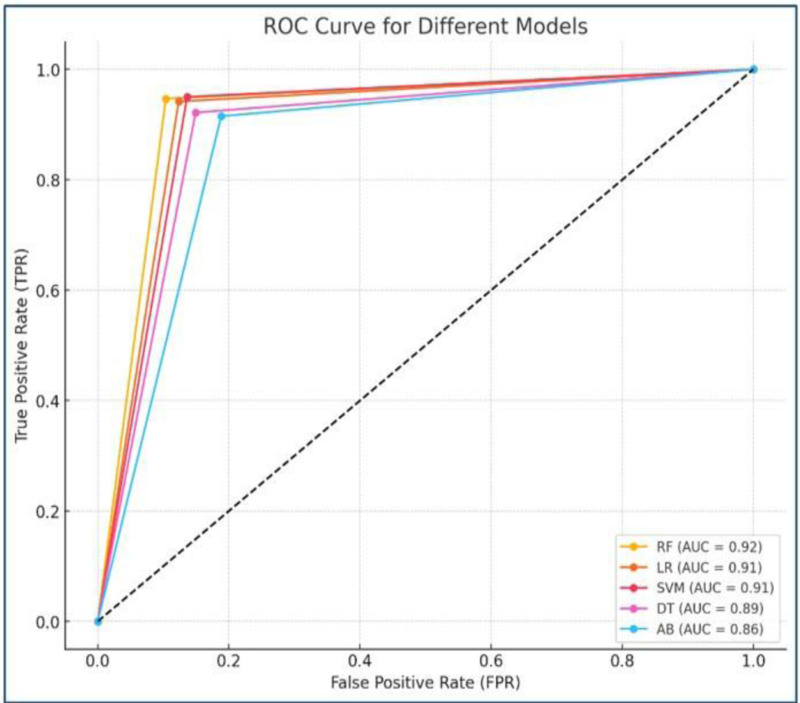
AUROC curve of default ML classifiers.

Further analyzed the area under the receiver operating characteristic (AUROC) curve for this experiment. It is a critical performance metric parameter used for evaluating binary classification models because it measures how well the model separates positive and negative classes, regardless of a specific classification threshold, and is robust to imbalanced data. This makes it an effective measure for assessing a model’s ability to distinguish between classes, even in real-world scenarios with uneven data distribution. Fig 9 depicts the AUROC curves for various default ML models. The RF reigns supreme with an AUROC of 0.92, signifying its exceptional ability to differentiate between thyroid and normal classes. LR and SVM model achieved comparable performance with high AUROC values of 0.91. DT demonstrated slightly superior performance with an AUROC of 0.89. While AB had a lower AUROC of 0.86, it still outperformed a random classification model.

#### Experiments 2: proposed hybrid GA-ML models.

**Fig 10:**
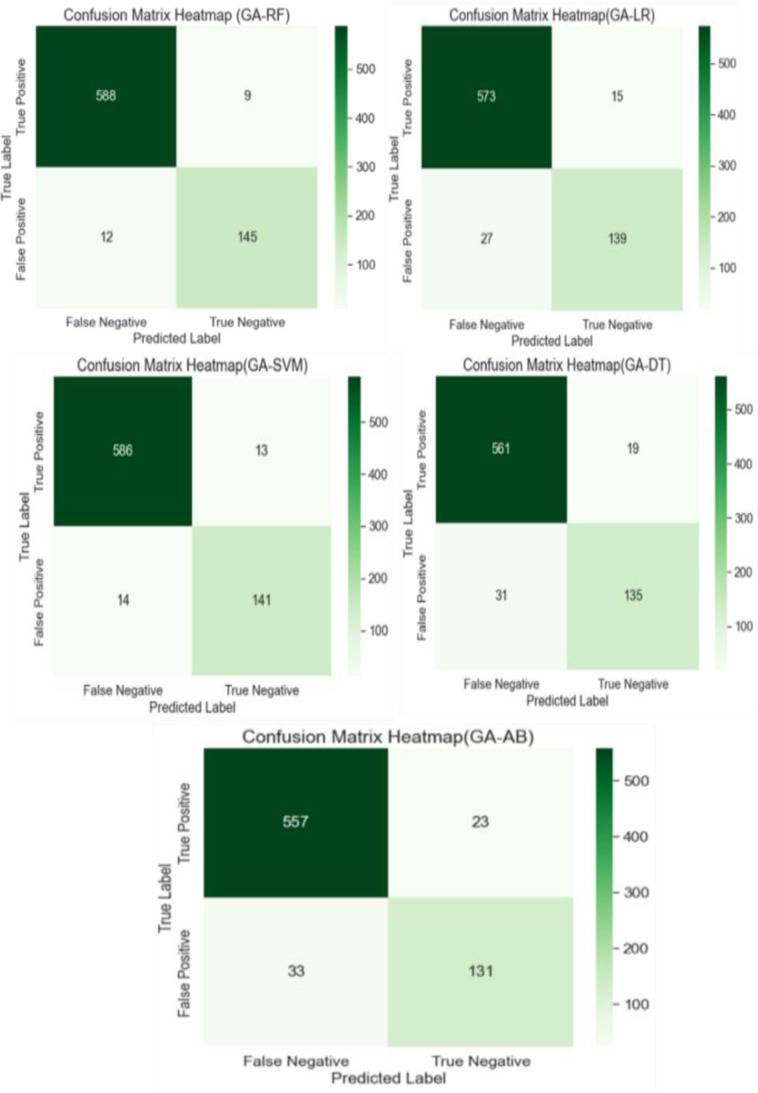
Performance metrics of GA-ML models.

**Fig 11:**
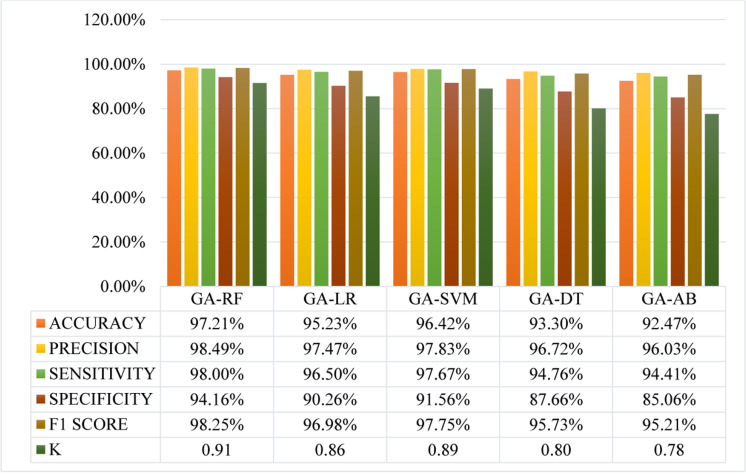
Performance metrics of proposed GA-ML classifiers.

In the second experiment, all the ML algorithms hybridized with GA for feature optimization, i.e., (GA-RF, GA-LR, GA-SVM, GA-DT, and GA-AB) and trained using 80% of the data, corresponding to 3017 patients. Subsequently, these trained models were tested on the remaining 20% of the data, equivalent to 754 patients. The hyperparameters of all proposed models were optimized using GA. The optimal values for each model are: for GA-RF, n_estimators = 100, criterion = gini, min_samples_split = 2, min_samples_leaf = 1; for GA-LR, penalty = l2, C = 1.0, solver = liblinear, max_iter = 100; for GA-SVM, C = 1.0, kernel = rbf, gamma = scale; for GA-DT, criterion = gini, splitter = best, min_samples_split = 2, min_samples_leaf = 1; and for GA-AB, n_estimators = 50, learning_rate = 0.001. Fig 10 shows the confusion matrix of ML-GA models and Fig 11 summarizes the performance metrics of GA-ML models, i.e., GA-RF, GA-LR, GA-SVM, GA-DT, and GA-AB applied to the thyroid dataset. Proposed GA-ML models show improved performance over their non-GA counterparts, with GA-RF achieving the highest accuracy of 97.21%. Precision values are consistently strong across all GA-ML models, particularly with GA-RF exhibiting the highest precision at 98.49%, indicating effective identification of positive cases. Sensitivity scores are notably high for GA-RF (98.00%) and GA-SVM (97.67%), demonstrating their ability to detect true positives accurately. Specificity varies across models but remains robust overall, with GA-RF leading at 94.16%. F1 scores, reflecting a balance between precision and recall, are notably elevated across all GA-ML models, ranging from 95.21% (GA-AB) to 98.25% (GA-RF). Additionally, Cohen’s Kappa coefficient (K), which measures agreement beyond chance, indicates substantial to almost perfect agreement (ranging from 0.78 to 0.91) for the GA-ML models. Overall, GA-ML models outperform their non-GA counterparts across various metrics, with GA-RF demonstrating superior performance in accuracy, precision, sensitivity, specificity, F1 score, and Cohen’s Kappa coefficient, highlighting the effectiveness of integrating GA with ML on the thyroid dataset.

**Fig 12:**
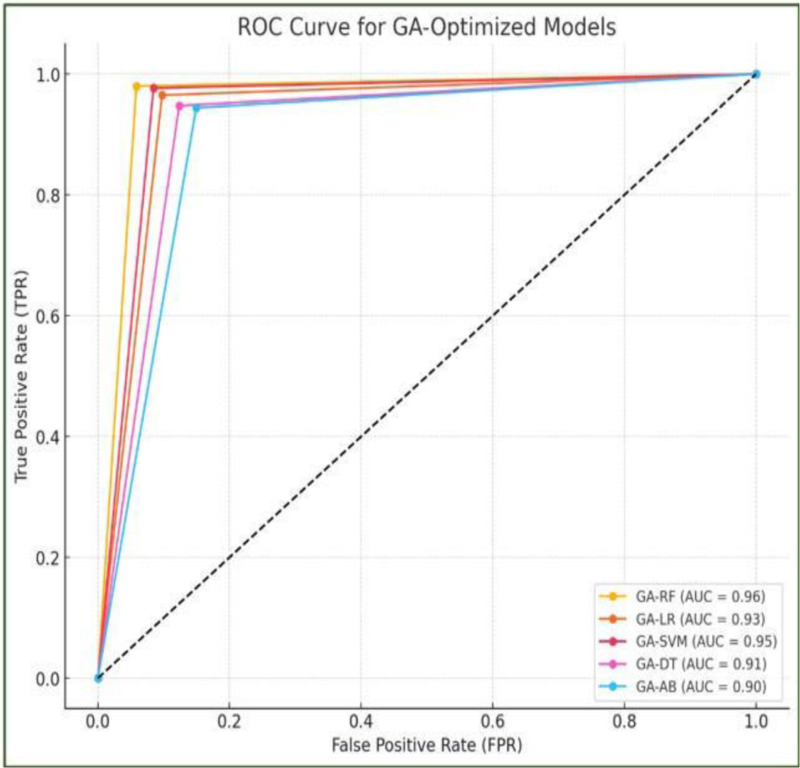
AUROC curve of proposed hybrid GA-ML models.

Further analyzed the AUROC curve of proposed models. Fig 12 depicts the AUROC curves for different hybrid GA-ML. Among these, GA-RF outperformed with an AUROC of 0.96, signifying its exceptional ability to differentiate between thyroid and normal classes. The GA-LR and GA-SVM models achieved comparable performance with high AUROC values of 0.93 and 0.95 respectively. GA-DT demonstrated slightly superior performance with an AUROC of 0.91. While AB had a lower AUROC of 0.90, it still outperformed a random classification model.

### Comparative analysis with previous research studies

#### Based on performance matrices.

In this section, the authors compared the results of the proposed models with default ML classifiers and existing research studies on thyroid classification as shown in Table 6. All studies, including the proposed approach, utilized the UCI thyroid disease dataset. The proposed GA-ML approach utilized a combination of GA with various ML algorithms including RF, LR, SVM, DT, and AB based on the results for default ML classifiers). The proposed GA-RF model achieved the highest accuracy (97.21%) compared to all default ML classifiers (ranging from 89.39% to 93.63%) and most existing studies (ranging from 87.5% to 95.87%). Existing studies explore a variety of algorithms including ANN [1,29,13], SVM [14,28], CNN [16], DT [30], LR [17,30], DT [44], CatBoost and XGBoost [29]. Studies [29] utilizing CatBoost and XGBoost achieved comparable accuracy (around 95%). However, these are ensemble methods that may be more complex than the proposed GA-RF model. The proposed GA-ML approach demonstrates a significant improvement in accuracy compared to both default ML models (without GA optimization) and existing studies, highlighting the effectiveness of GA for hyperparameter tuning and feature selection. While some existing studies achieve comparable accuracy using deep learning or ensemble methods, the proposed GA-RF model offers a potentially simpler and more interpretable approach with excellent performance. This analysis highlights the promising performance of the proposed GA-ML approach for thyroid disease classification.

**Table 6:** Comparison of proposed model with default ML classifiers and recent existing studies.

References	Dataset	Data Preprocessing and feature selection methods	Methodology	Accuracy (%)
Guleria et al. [1]	UCI	Missing value handled	ANN	93.826
Mir et al. [14]	UCI	Normalization	SVM	92.07
Zhang et al. [16]	Tertiary Hospital	Data Scaling	CNN	90.90
Duggal et al. [28]	UCI	Correlation	SVM	92.92
Islam et al. [29]	UCI	Normalization	ANN	95.87
			CatBoost	95.38
			XGBoost	95.33
Alyas et al. [13]	UCI	Missing value handled	ANN	94
Sankar et al. [30]	UCI	Normalization	DT	87.5
			LR	81.25
Chaubey et al. [44]	UCI	**----**	DT	87.5
Default ML Classifiers	UCI	Missing value, Data scaling, Correlation	RF	93.63
			LR	92.84
			SVM	93.24
			DT	90.72
			AB	89.39
Proposed Approaches (GA-ML)	UCI	Missing value, Data scaling, Correlation, Feature selection and GA	GA-RF	97.21

#### Based on time complexity.

The evaluation of time complexity, comparing the proposed GA-ML frameworks with previous GA-based studies highlights the significant advantages of the proposed models in terms of classification accuracy and computational efficiency which is hidden in Table 7. For instance, GA-RF achieved the highest accuracy of 97.21% with a relatively low execution time of 121.07 seconds showcasing its robustness and scalability. Similarly, GA-LR (95.23%, 101.61s) and GA-SVM (96.42%, 117.24s) demonstrate an optimal trade-off between predictive performance and computational cost. In contrast conventional approaches such as GA-CNN and Traditional GA require significantly higher execution times, reaching 9158.88 seconds while delivering relatively lower accuracy. These results underscore the effectiveness of the proposed GA-ML models in achieving efficient algorithmic convergence. These findings make them ideal for real-world, performance-intensive scenarios.

**Table 7:** Time complexity comparison with existing studies.

Approaches	Classification Accuracy (%)	Number of generations	Time (Second)
GA-CNN	86.39	100	9158.88
GAPSO-RF	87.80	30	2559.10
GA-NB	83.68	100	111.41
Traditional GA	87.05	100	7799.98
GA-SVM	88.34	100	148.05
**Proposed GA-ML**			
**GA-RF**	**97.21**	**100**	**121.07**
**GA-LR**	**95.23**	**100**	**101.61**
**GA-SVM**	**96.42**	**100**	**117.24**
**GA-DT**	**93.30**	**100**	**107.97**
**GA-AB**	**92.47**	**100**	**113.53**

### Main findings of the proposed hybrid GA-ML models

This study investigated the effectiveness of various ML models for thyroid disease classification. The authors evaluated both default models (in experiment 1) and models optimized with a meta-heuristic GA approach (in experiment 2). The initial experiment utilizing default models (RF, LR, SVM, DT, AB) highlighted the need for further optimization. While all models achieved a baseline level of performance, they exhibited limitations, particularly in balancing precision and recall. This suggests the potential for improvement through hyperparameter tuning and feature selection. The second experiment addressed these limitations by incorporating a GA approach for hyperparameter tuning and feature selection. The proposed GA-ML models, i.e., GA-RF, GA-LR, GA-SVM, GA-DT, and GA-AB demonstrated significant improvement compared to their default counterparts. Notably, GA-RF emerged as the top performer, achieving superior accuracy, precision, sensitivity, and F1 score. This improvement can be attributed to the GA’s ability to optimize hyperparameters and select a subset of 23 optimal features from the original 30 features. This feature reduction not only improved model performance but also reduced complexity, making the proposed models more efficient. The enhanced performance of GA-ML models, particularly GA-RF, translates to a greater ability to distinguish between positive and negative thyroid cases. This improved accuracy and reliability make GA-ML models a potentially appropriate approach for clinical applications. The high F1 score of GA-RF suggests a good balance between identifying true positives and minimizing false positives, which is crucial for accurate diagnosis and patient care.

## Conclusion

This study highlights the efficacy of ML techniques in the diagnosis of thyroid disorders, specifically hypothyroidism and hyperthyroidism. By employing five different ML algorithms, i.e., LR, RF, SVM, AB, and DT in conjunction with a GA for feature selection, the authors aimed to enhance diagnostic overall performance using the UCI thyroid dataset. The preprocessing steps, which included addressing missing data, performing data scaling, and assessing feature correlations, were crucial for preparing the dataset for effective analysis. Two sets of experiments were conducted: one with default ML classifiers and another with the proposed hybrid GA-ML models. Performance metrics such as Accuracy, F1 Score, Specificity, Sensitivity, Cohen’s Kappa, and Precision were used to evaluate the models. The results demonstrated that the hybrid GA-RF model achieved the highest accuracy of 97.21%, outperforming not only the default ML classifiers but also the hybrid models and previous studies. This indicates that the integration of GA for feature selection significantly enhances the predictive performance of the ML models. The proposed GA-RF model offers a promising approach for the rapid and precise diagnosis of thyroid disorders. The combination of advanced preprocessing techniques and the hybrid ML approach underscores the potential of ML in medical diagnostics, providing a reliable and efficient alternative to traditional manual analysis. This study paves the way for further research into the application of ML techniques in other areas of medical diagnosis, where rapid and accurate decision-making is critical.

## Limitations and future work recommendations

This study offers valuable insights into utilizing GA-ML models for thyroid disease classification. However, there are limitations to consider and avenues for future exploration. The study relies on the UCI thyroid dataset, which might have limitations in size and diversity compared to real-world clinical datasets. The research employed Stratified K-Fold Cross-Validation, a robust internal validation technique. However, further validation on a completely independent (external or new) dataset would strengthen the model’s reliability. By addressing these limitations in future research could involve collaborating with medical professionals to integrate GA-ML models into clinical workflows and assess their effectiveness in real-world datasets for more comprehensive analysis [36].

## References

[1] Guleria K, Sharma S, Kumar S, Tiwari S. Early prediction of hypothyroidism and multiclass classification using predictive machine learning and deep learning. Measurement: Sensors. 2022;24:100482. doi: 10.1016/j.measen.2022.100482

[2] Butchi Raju K, Kumar Lakineni P, Indrani KS, Mary Swarna Latha G, Saikumar K. Optimized building of machine learning technique for thyroid monitoring and analysis. In: 2021 2nd International Conference on Smart Electronics and Communication (ICOSEC), 2021. doi: 10.1109/icosec51865.2021.9591814

[3] Hu M, Asami C, Iwakura H, Nakajima Y, Sema R, Kikuchi T, et al. Development and preliminary validation of a machine learning system for thyroid dysfunction diagnosis based on routine laboratory tests. Commun Med (Lond). 2022;2:9. doi: 10.1038/s43856-022-00071-1 35603277 PMC9053267

[4] Keleş A, Keleş A. ESTDD: Expert system for thyroid diseases diagnosis. Expert Systems with Applications. 2008;34(1):242–6. doi: 10.1016/j.eswa.2006.09.028

[5] Alnaggar í µí M, Handosa í µí M, Medhat T, Rashad í µí MZ, Author C, Alnaggar M. Thyroid Disease Multi-class Classification based on Optimized Gradient Boosting Model. Online, 2023. Online. Available from: https://ejai.journals.ekb.eg

[6] Riajuliislam M, Rahim KZ, Mahmud A. Prediction of Thyroid Disease(Hypothyroid) in Early Stage Using Feature Selection and Classification Techniques. In: 2021 International Conference on Information and Communication Technology for Sustainable Development (ICICT4SD), 2021. pp. 60–64. Available from:10.1109/icict4sd50815.2021.9397052

[7] Mohan S, Thirumalai C, Srivastava G. Effective Heart Disease Prediction Using Hybrid Machine Learning Techniques. IEEE Access. 2019;7:81542–54. doi: 10.1109/access.2019.2923707

[8] Tajvidi Asr C, Rahimi R, Pourasad MH, Zayer MH, Momenzadeh S, Ghaderzadeh MR. Hematology and hematopathology insights powered by machine learning: shaping the future of blood disorder management. 2024. Available from: https://ijbc.ir

[9] Ghaderzadeh M, Shalchian A, Irajian G, Sadeghsalehi H, Zahedi bialvaei A, Sabet B. Artificial Intelligence in Drug Discovery and Development Against Antimicrobial Resistance: A Narrative Review. Iran J Med Microbiol. 2024;18(3):135–47. doi: 10.30699/ijmm.18.3.135

[10] Ahmadirad Z. The role of AI and machine learning in supply chain optimization. 2025. doi: 10.63053/ijset.77

[11] Dwivedi AK. Performance evaluation of different machine learning techniques for prediction of heart disease. Neural Comput & Applic. 2016;29(10):685–93. doi: 10.1007/s00521-016-2604-1

[12] Dhanka S, Maini S. Multiple Machine Learning Intelligent Approaches for the Heart Disease Diagnosis. In: IEEE EUROCON 2023 - 20th International Conference on Smart Technologies, 2023. 147–52. Available from: doi: 10.1109/eurocon56442.2023.10199080

[13] Alyas T, Hamid M, Alissa K, Faiz T, Tabassum N, Ahmad A. Empirical Method for Thyroid Disease Classification Using a Machine Learning Approach. Biomed Res Int. 2022;2022:9809932. doi: 10.1155/2022/9809932 35711517 PMC9197629

[14] Mir YI, Mittal S. Thyroid disease prediction using hybrid machine learning techniques: An effective framework. International Journal of Scientific & Technology Research. 2020;9:2.

[15] Yeh W-C. Novel swarm optimization for mining classification rules on thyroid gland data. Information Sciences. 2012;197:65–76. doi: 10.1016/j.ins.2012.02.009

[16] Zhang X, Lee VCS, Rong J, Lee JC, Song J, Liu F. A multi-channel deep convolutional neural network for multi-classifying thyroid diseases. Computers in Biology and Medicine. 2022;148:105961. doi: 10.1016/j.compbiomed.2022.105961 35985185

[17] Butchi Raju K, Kumar Lakineni P, Indrani KS, Mary Swarna Latha G, Saikumar K. Optimized building of machine learning technique for thyroid monitoring and analysis. In: 2021 2nd International Conference on Smart Electronics and Communication (ICOSEC), 2021. 1–6. Available from: doi: 10.1109/icosec51865.2021.9591814

[18] Rasitha G, Fphtm BF. A role of decision tree classification data mining technique in diagnosing thyroid disease. 2016.

[19] Khan F, Ayoub S, Gulzar Y, Majid M, Reegu FA, Mir MS, et al. MRI-Based Effective Ensemble Frameworks for Predicting Human Brain Tumor. J Imaging. 2023;9(8):163. doi: 10.3390/jimaging9080163 37623695 PMC10455878

[20] Anand V, Gupta S, Gupta D, Gulzar Y, Xin Q, Juneja S, et al. Weighted Average Ensemble Deep Learning Model for Stratification of Brain Tumor in MRI Images. Diagnostics (Basel). 2023;13(7):1320. doi: 10.3390/diagnostics13071320 37046538 PMC10093740

[21] Khan F, Gulzar Y, Ayoub S, Majid M, Mir MS, Soomro AB. Least square-support vector machine based brain tumor classification system with multi model texture features. Front Appl Math Stat. 2023;9. doi: 10.3389/fams.2023.1324054

[22] Singh LK, Khanna M, Monga H, singh R, Pandey G. Nature-Inspired Algorithms-Based Optimal Features Selection Strategy for COVID-19 Detection Using Medical Images. New Gener Comput. 2024;42(4):761–824. doi: 10.1007/s00354-024-00255-4

[23] Khanna M, Singh LK, Shrivastava K, Singh R. An enhanced and efficient approach for feature selection for chronic human disease prediction: A breast cancer study. Heliyon. 2024;10(5):e26799. doi: 10.1016/j.heliyon.2024.e26799 38463826 PMC10920178

[24] Singh LK, Khanna M, Garg H, Singh R. Efficient feature selection based novel clinical decision support system for glaucoma prediction from retinal fundus images. Med Eng Phys. 2024;123:104077. doi: 10.1016/j.medengphy.2023.104077 38365344

[25] Kumar Singh L, Khanna M, singh R. A novel enhanced hybrid clinical decision support system for accurate breast cancer prediction. Measurement. 2023;221:113525. doi: 10.1016/j.measurement.2023.113525

[26] Singh LK, Khanna M, Thawkar S, Singh R. Collaboration of features optimization techniques for the effective diagnosis of glaucoma in retinal fundus images. Advances in Engineering Software. 2022;173:103283. doi: 10.1016/j.advengsoft.2022.103283

[27] Pal R, Anand T, Dubey SK. Evaluation and performance analysis of classification techniques for thyroid detection. doi: 10.1504/ijbis.2018.091862

[28] Duggal P, Shukla S. Prediction Of Thyroid Disorders Using Advanced Machine Learning Techniques. In: 2020 10th International Conference on Cloud Computing, Data Science & Engineering (Confluence), 2020. 670–5. Available from: doi: 10.1109/confluence47617.2020.9058102

[29] Islam SS, Haque MS, Miah MSU, Sarwar TB, Nugraha R. Application of machine learning algorithms to predict the thyroid disease risk: an experimental comparative study. PeerJ Comput Sci. 2022;8:e898. doi: 10.7717/peerj-cs.898 35494828 PMC9044232

[30] Sankar S, Potti A, Chandrika GN, Ramasubbareddy S. Thyroid Disease Prediction Using XGBoost Algorithms. JMM. 2022. doi: 10.13052/jmm1550-4646.18322

[31] Quinlan R. UCI Thyroid dataset. University of California Irvine (UCI) ML repository. 1987. doi: 10.24432/C5D010 Available from: https://archive.ics.uci.edu/dataset/102/thyroid%20disease

[32] Kumar Y, Koul A, Sisodia PS, Shafi J, Verma K, Gheisari M, et al. Heart Failure Detection Using Quantum‐Enhanced Machine Learning and Traditional Machine Learning Techniques for Internet of Artificially Intelligent Medical Things. Wireless Communications and Mobile Computing. 2021;2021(1). doi: 10.1155/2021/1616725

[33] Kumar Dubey A, Choudhary K, Sharma R. Predicting Heart Disease Based on Influential Features with Machine Learning. Intelligent Automation & Soft Computing. 2021;30(3):929–43. doi: 10.32604/iasc.2021.018382

[34] Song Y-Y, Lu Y. Decision tree methods: applications for classification and prediction. Shanghai Arch Psychiatry. 2015;27(2):130–5. doi: 10.11919/j.issn.1002-0829.215044 26120265 PMC4466856

[35] Dhanka S, Maini S. HyOPTXGBoost and HyOPTRF: Hybridized Intelligent Systems using Optuna Optimization Framework for Heart Disease Prediction with Clinical Interpretations. Multimed Tools Appl. 2024;83(29):72889–937. doi: 10.1007/s11042-024-18312-x

[36] Absar N, Das EK, Shoma SN, Khandaker MU, Miraz MH, Faruque MRI, et al. The Efficacy of Machine-Learning-Supported Smart System for Heart Disease Prediction. Healthcare. 2022;10(6):1137. doi: 10.3390/healthcare10061137 35742188 PMC9222326

[37] Fan Y, Bai J, Lei X, Zhang Y, Zhang B, Li K-C, et al. Privacy preserving based logistic regression on big data. Journal of Network and Computer Applications. 2020;171:102769. doi: 10.1016/j.jnca.2020.102769

[38] Qureshi SA, Hsiao WW-W, Hussain L, Aman H, Le T-N, Rafique M. Recent Development of Fluorescent Nanodiamonds for Optical Biosensing and Disease Diagnosis. Biosensors (Basel). 2022;12(12):1181. doi: 10.3390/bios12121181 36551148 PMC9775945

[39] Chiroma H, Noor ASM, Abdulkareem S, Abubakar AI, Hermawan A, Qin H, et al. Neural Networks Optimization through Genetic Algorithm Searches: A Review. Appl Math Inf Sci. 2017;11(6):1543–64. doi: 10.18576/amis/110602

[40] Mathew TV. Genetic Algorithm. Report submitted at IIT Bombay. 2012;53.

[41] Dhanka S, Maini S. Random Forest for Heart Disease Detection: A Classification Approach. In: 2021 IEEE 2nd International Conference On Electrical Power and Energy Systems (ICEPES), 2021. 1–3. doi: 10.1109/icepes52894.2021.9699506

[42] Dhanka S, Bhardwaj VK, Maini S. Comprehensive analysis of supervised algorithms for coronary artery heart disease detection. Expert Systems. 2023;40(7). doi: 10.1111/exsy.13300

[43] Sharma A, Dhanka S, Kumar A, Maini S. A comparative study of heterogeneous machine learning algorithms for arrhythmia classification using feature selection technique and multi-dimensional datasets. Eng Res Express. 2024;6(3):035209. doi: 10.1088/2631-8695/ad5d51

[44] Chaubey G, Bisen D, Arjaria S, Yadav V. Thyroid Disease Prediction Using Machine Learning Approaches. Natl Acad Sci Lett. 2020;44(3):233–8. doi: 10.1007/s40009-020-00979-z

