# Expression of Concern: Comprehensive framework for thyroid disorder diagnosis: Integrating advanced feature selection, genetic algorithms, and machine learning for enhanced accuracy and other performance matrices

**DOI:** 10.1371/journal.pone.0343473

**Published:** 2026-02-23

**Authors:** The PLOS One Editors

After this article [1] was published, the following concerns were noted:

- Potential non-compliance with the PLOS Authorship policy.
- The article cited as Reference 13 was retracted before [1] was published.
- The articles cited as References 35, and 41–43 do not appear to support the corresponding cited statement.

The authors stated that the retracted Reference 13 was included in the manuscript before it was retracted, and the retraction was not identified prior to publication of [1]. They requested that this reference be removed from [1]. The Editors recognize that the cited reference does not impact the reliability of the study.

The authors asserted that the References 35, and 41–43, which are all co-authored by authors of [1], are relevant to the methodological framework of this study and demonstrate their research group’s expertise in similar research topics.

In light of the cumulative issues and concerns, which were not resolved in our discussions with the authors, the *PLOS One* Editors issue this Expression of Concern.
